# A robotic wheelchair trainer: design overview and a feasibility study

**DOI:** 10.1186/1743-0003-7-40

**Published:** 2010-08-13

**Authors:** Laura Marchal-Crespo, Jan Furumasu, David J Reinkensmeyer

**Affiliations:** 1 Mechanical and Aerospace Engineering Department, University of California, Irvine, CA, USA; 2 Rehabilitation Engineering Research Center on Technology for Children With Orthopedic Disabilities, Rancho Los Amigos National Rehabilitation Center, Downey, CA, USA

## Abstract

**Background:**

Experiencing independent mobility is important for children with a severe movement disability, but learning to drive a powered wheelchair can be labor intensive, requiring hand-over-hand assistance from a skilled therapist.

**Methods:**

To improve accessibility to training, we developed a robotic wheelchair trainer that steers itself along a course marked by a line on the floor using computer vision, haptically guiding the driver's hand in appropriate steering motions using a force feedback joystick, as the driver tries to catch a mobile robot in a game of "robot tag". This paper provides a detailed design description of the computer vision and control system. In addition, we present data from a pilot study in which we used the chair to teach children without motor impairment aged 4-9 (n = 22) to drive the wheelchair in a single training session, in order to verify that the wheelchair could enable learning by the non-impaired motor system, and to establish normative values of learning rates.

**Results and Discussion:**

Training with haptic guidance from the robotic wheelchair trainer improved the steering ability of children without motor impairment significantly more than training without guidance. We also report the results of a case study with one 8-year-old child with a severe motor impairment due to cerebral palsy, who replicated the single-session training protocol that the non-disabled children participated in. This child also improved steering ability after training with guidance from the joystick by an amount even greater than the children without motor impairment.

**Conclusions:**

The system not only provided a safe, fun context for automating driver's training, but also enhanced motor learning by the non-impaired motor system, presumably by demonstrating through intuitive movement and force of the joystick itself exemplary control to follow the course. The case study indicates that a child with a motor system impaired by CP can also gain a short-term benefit from driver's training with haptic guidance.

## Introduction

Independent mobility is crucial for children' cognitive, emotional, and psychosocial development [1-5]. Providing a child with self-controlled, powered mobility provides motivation for learning since the chair becomes a tool for exploration, locomotion, and play. However, many children with disabilities do not achieve independent mobility, especially at a young age, when this stimulus of mobility particularly influences development. It seems likely that this situation is caused in part by limited training time: children with severe disabilities can and do learn new motor skills, but often more slowly than children without developmental disorders. Because the conventional approach for powered wheelchair driver's training is expensive and labor-intense, typically requiring the hand-over-hand assistance of a skilled therapist to facilitate learning and ensure safety during training sessions, children who do not learn quickly may experience limited training time, preventing them from achieving independent driving ability.

To lower the cost and improve accessibility to training, we have developed a robotic powered wheelchair system on which young children with a disability can safely develop driving skills at their own pace with minimum assistance from a therapist. We equipped a powered wheelchair with a web-cam that identifies and tracks a line on the floor to achieve a self steering function along a training course. We added a force-feedback joystick to implement an algorithm [6] that can demonstrate (through movement and force of the joystick itself) exemplary control to follow the course, while systematically modulating the strength and sensitivity of such haptic demonstration, making the joystick stiffer (and more damped) when more assistance is needed. This method gradually exposes the child to the dynamics of a normal powered wheelchair, in an analogous fashion to bicycle training wheels. The idea is to let the individual learn from the experience of making errors repeatedly and safely in a structured environment, while reducing demands on the supervising caregiver.

The smart powered wheelchair described here is intended to work as a tool targeted specifically at driver's training, in contrast to most other pediatric smart wheelchairs developed to the date (e.g. [1,7,8]), which aim to help children with disabilities to steer a power wheelchair during activities of daily living by relieving some of the control burden. The pediatric smart wheelchair developed at the CALL Center of the University of Edinburgh, Scotland [8] is a relevant example to our work. This institution has developed a pediatric smart wheelchair trainer with bump sensors, sonar sensors, and the ability to follow tape lines on the floor to train disabled children drivers to improve their mobility using different levels of autonomy. However, the CALL Center smart wheelchair does not provide haptic feedback while following the line on the floor. A primary design goal of the system described here was to have it gradually and automatically give more control to the child as learning progresses, rather than "take over" control. Our working hypothesis is that by appropriately challenging the child, the development of steering skill will be facilitated, a hypothesis consistent with the Challenge Point Theory from motor learning research [9].

To intelligently challenge the user, the chair uses fading, haptic guidance. Haptic guidance is a motor-training strategy in which a trainer physically interacts with the participant's limbs during movement training, steering them along desired movements [10-13]. Haptic guidance is commonly used by rehabilitation therapists in wheelchair driver's training, as well as in many other rehabilitation and sports training applications. Besides providing a safety benefit, a common concept is that physically demonstrating a movement may help people learn how to perform it. However, there is little evidence that robotic guidance is beneficial for human motor learning beyond enhancing safety, compared to unassisted practice. The long-standing "Guidance Hypothesis" in fact asserts that providing too much physical or cognitive assistance during training will impair learning, because it obviates the nervous system from learning the error-correction strategies required to successfully perform the target task [14,15]. A number of studies have confirmed this hypothesis, finding that physically guiding movements does not aid motor learning and may in fact hamper it [10-13,16-21].

Thus, a concern we had at the onset with the approach presented here is that, while providing haptic guidance could make training safer and help automate training, it may impair learning of driving skill. To address this concern, we performed preliminary studies with a virtual reality wheelchair driving simulator and non-impaired, adult subjects [6,22]. We developed a control algorithm to provide haptic guidance with a force feedback steering wheel as a person steers a simulated power wheelchair. We incorporated a novel guidance-as-needed strategy, which adjusts levels of guidance based on the ongoing performance of the driver. Preliminary studies from our lab showed that training with guidance-as-needed improved the drivers' steering ability more than training without guidance, apparently because it helped learn when to begin turns [6]. Furthermore, training with haptic guidance was more beneficial for initially less skilled people [22].

These previous studies were done with a virtual wheelchair that moved at a constant speed, with a force feedback steering wheel, and with adult participants. As described in this paper, we have now implemented the steering algorithm using a force feedback joystick on a pediatric wheelchair. This necessitated development of a computer vision system, as well as an extension of the haptic guidance algorithm to take into account changes in wheelchair velocity. To determine if the resulting robotic wheelchair trainer could assist effectively in training, we performed an experiment with 22 non-disabled children (aged 3-9, mean 6.6 ± .5 SD) randomly assigned into "Guidance" and "No Guidance" groups. We compared the resulting performance after training with guidance and training without assistance in a single training session in order to determine if robotic guidance promotes learning compared to training without guidance for the non-injured, developing human motor system. We also report the results of a case study with one 8-year-old child with a severe motor impairment due to cerebral palsy, who replicated the Guidance single-session training protocol. We compared her increase of steering ability with the "Guidance" and "No Guidance" groups to determine if a impaired motor system can also benefit from haptic guidance during driver's training.

## Methods

### The smart power wheelchair system

**Figure 1:**
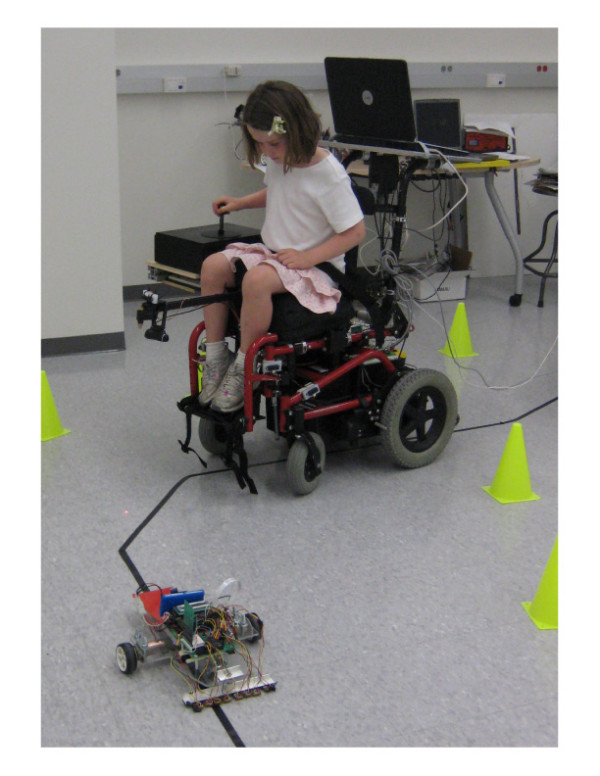
**ROLY -RObot-assisted Learning for Young drivers**. We developed a robotic wheelchair trainer that steers itself along a course marked by a line on the floor using computer vision, haptically guiding the child's hand in appropriate steering motions using a force feedback joystick. The child is instructed to follow the line with a spot of light from a laser pointer mounted on the chair, creating the smallest amount of error possible. To motivate the children during training, we programmed a small mobile robot to follow the same black line, and requested the child to try to catch it in a game of "robot tag".

We developed a prototype pediatric smart wheelchair (ROLY -RObot-assisted Learning for Young drivers) that incorporates a webcam to achieve a self steering function along a training course (defined by a black line on the floor), and a force-feedback joystick to implement an algorithm that can demonstrate (through movement and force of the joystick itself) exemplary control to follow the course, while systematically modulating the strength and sensitivity of such haptic demonstration (Figure 1). We installed the camera, joystick and a laptop on a commercial pediatric powered wheelchair (Quickie Z-500). The force-feedback joystick (Figure 1, Immersion Impulse Stick) uses electric motors that can be programmed to produce forces up to 14.5 N (3.5 lbf), and can move to a desired position with a resolution of 0.01 degrees. The joystick can physically demonstrate the control motion required for successful driving along the test course, applying forces to the participants' hands only when s/he makes steering errors, and thus correct the joystick motion to bring the power wheelchair back to the desired circuit. The stiffness and damping effects of the force-feedback joystick can be modified, thus making the joystick stiffer (and more damped) when more assistance is needed.

The guidance provided by the joystick was designed to anticipate turns, as is described in previous work [6]. As a wheelchair is a non-holomonic vehicle, in order to minimize the tracking error when turning, the driver has to start the movement before the track changes direction. The driving action is then dependent on what the driver sees in front of him or her. We translated this look-ahead idea to the guidance controller, similarly to Sheridan's work in constrained preview control [23], using the distance and direction error with respect to a point situated a determined distance *d *ahead of the vehicle. We also incorporated previous findings [6,13,22] in motor learning through the implementation of a faded control algorithm that changes the "firmness" of the guidance as the participants perform the task, limiting large errors, while being constantly presented with a higher degree of challenge. The guidance controller had the following form:

(1) $$Jx_{des}=Kd\cdot e_{dis}+Ka\cdot e_{ang}+Ba\cdot \frac{d(e_{ang})}{dt}.$$

The desired joystick x-axis position (*Jx_des_*) depends on the look-ahead distance error (*e_dis_*), the look-ahead direction error (*e_ang_*), and its time derivative (*d*(*e_ang_*)/*dt*). The guidance was defined as a force that the joystick applies on the child's hands. Note that only the steering command is controlled (x-axis), while the wheelchair speed (y-axis) was freely selected by the driver during the experiment. The guidance force (*F_assist_*) was calculated as follows:

(2) $$F_{\text{a}ssist}=Kj\cdot (Jx-Jx_{des})+Bj\cdot \frac{d(Jx)}{dt}$$

Where *K_j _*and *B_j _*are the joystick's stiffness and damping coefficients, which can be modulated through the DirectX force feedback (FFB) libraries, and *Jx *is the current x-axis joystick position. It is clear that as the wheelchair's position and direction errors become larger, the desired joystick x-axis position (*Jx_des_*) and the joystick position error (*Jx - Jx_des_*) increase, and thus the guidance force (*F_assist_*) becomes larger. Note however, that at equal errors, when the stiffness and damping coefficients (*K_j _*and *B_j_*) are larger, the guidance force will be larger.

We faded the firmness of the force feedback allowing more freedom (more error) around the line as training progressed, but always limiting large errors. In other words, as the participant drove, the joystick applied less force for the same error values by updating the stiffness and damping control gains (*K_j _*and *B_j_*).

(3) $$G_{i+1}=f_{R}\cdot G_{i}$$

where *G *represents the value of the control gains, *f_R _*is the "forgetting factor" (*f_R _*= 0.9976), and the subscript *i *indicates the *i - th *iteration. Note that the forgetting factor *f_R _*must be less than 1 in order to decrease the value of the guidance as training proceeds; the particular value chosen was selected to decrease guidance exponentially with a time constant of 4.63 minutes.

We observed in preliminary experiments with experienced drivers that their look-ahead distance was linearly dependent on the speed: as the wheelchair moves faster, they needed a larger look-ahead distance to correctly react to the sudden changes of line direction, and steer accurately with minimal tracking error. We ran several trials with the chair at different speeds and found a linear correlation between the optimal look-ahead distance and the power wheelchair speed that allows the wheelchair to steer accurately at different speeds, where the optimal look-ahead distance is defined as the look-ahead distance that minimizes the overall tracking error in a trial, when the wheelchair steers autonomously:

(4) d=−80·Jy+160

where *d *is the optimal look-ahead distance in image coordinates, and *Jy *is the y-axis position of the joystick handler (ranging from 0 to 1). Thus, as the wheelchair moves faster, the computer vision system calculates the look-ahead errors using a greater look-ahead distance. The maximum wheelchair speed (*y - axis *= 1) in longitudinal direction is 1.28 m/s, and the maximum mean speed through the circuit is 0.38 m/s.

#### Sensor systems

To calculate the appropriate steering assistance forces, the smart wheelchair has to know the look-ahead error (*e_dis_*). We developed an on-board vision system that uses a low-cost webcam (Figure 1, QuickCam Pro 9000) mounted at the front of the wheelchair and implemented a line-following algorithm on the laptop using Simulink. The vision system algorithm identifies the black line in the video stream using color classification, edge detection algorithms and the Hough transformation, tracks the line using a Kalman filter, and calculates the look-ahead distance of the wheelchair to the line (*e_dis_*), and the direction of the wheelchair with respect to the line (*e_ang_*) using inverse perspective mapping, as a continuous variable no matter what the wheelchair's position.

**Figure 2:**
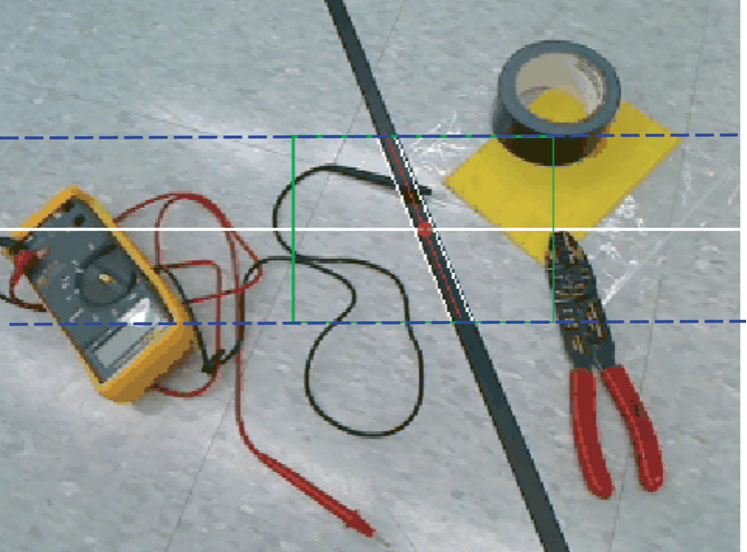
**Image from the camera**. The image from the camera is 240 × 320, however we define a region of interest (ROI) of 80 × 320 (area between dashed blue lines) around the look-ahead distance (depicted as a horizontal white line). The final ROI is calculated through tracking algorithms and depicted as a square around the line.

The vision system algorithm is fed with 240 × 320 greyscale frames. However, to reduce computational time, we further reduced the size of the region of interest (ROI) to 40 pixels above and below the look-ahead position (represented as a horizontal white line on Figure 2). The greyscale ROI was then converted into a black and white image (BW), such that pixels in the ROI with an intensity value below a threshold (*I *= 0.3) were considered as candidate points to be part of the line (candidate points = black). We defined two 2 D FIR filters to detect vertical left and right edges in the new BW frame and applied the Hough transform to the filtered images (one per each left and right edges) to seek potential lines' edges. In order to overcome noise problems created by the wheelchair's continuous movement, we designed a robust tracking system that uses two Kalman filters (one per each left and right line edges), and a parameter classification algorithm, able to determine if the two edges of a candidate line are indeed the edges of the course line, based on the distance between edges. The desired ROI is then further reduced to 40 pixels to the sides of the detected line (depicted as a square in Figure 2). When the candidate edges are classified as "no line", the ROI is increased by 5 pixels to the sides at each sample time until a correct tracking line is detected.

The camera was mounted in front of the wheelchair and tilted with respect to vertical, and thus images from the webcam were perturbed by perspective: parallel lines in the real world appeared as converging lines in the image plane. We restored the image to its original undistorted 3-D coordinates, which required the knowledge of the camera parameters, such as height, tilt angle, and focal length, which were calculated through the camera calibration [24].

In order to control the wheelchair movement, the power wheelchair has a low level controller (Pilot+, Penny & Giles) that translates the signals sent by the default commercial joystick into the two independent electrical motors/brakes. In order for our central computer to communicate with the Pilot+ controller, we added an OMNI+ interface which accepts signals from many different types of input devices (such as analog joysticks, and 5 switch input devices) and translates them into commands compatible with the Pilot+ controller. The analog signals required to be translated through the OMNI+ special interface are computer generated, and are generally proportional to the position of the joystick handler. The pseudo analog joystick signals are converted to analog signals through a low-cost A/D card (Labjack U12) at up to 50 Hz per channel.

For additional safety, we incorporated five low cost infrared proximity detectors (Sharp GP2D120), on the front of the wheelchair. These sensors take continuous distance readings and send them to an Arduino Diecimila minicontroller which sends a digital signal to the OMNI+ interface when an obstacle is detected in order to safely stop the wheelchair.

### The Driving Task: Robot Tag

To motivate the children during training, we programmed a small mobile robot to follow the same black line on the floor, and requested the child to try to catch it in a game of "robot tag". If a child steered off the black line, trying to take a shortcut, the smart wheelchair halved its speed, whereas the speed of the small robot was kept constant (controllable through a remote). We also vibrated the joystick, to reinforce the acquisition of the cause-effect relationship between the drive cutting the corners and the wheelchair slowing down, and the joystick vibrating. However, we note that the joystick vibration is a kind of haptic assistance input. Thus, when practicing without assistance, both haptic guidance, and haptic vibration sensory inputs were disabled.

The small robot is caught when the wheelchair vision system detects the red tag on the small robot (Figure 1) through *Y'CbCr *color segmentation. When the robot is caught, the wheelchair stops for 10 seconds, plays an amusing sound on the laptop, and sends a signal to the small robot through a wireless transmitter, which makes the small robot stop and perform a funny "dance" while beeping.

### Ergonomic modifications to account for a child with CP

We slightly modified the smart wheelchair system to account for the child with special needs due to cerebral palsy. Specifically, we located the camera overhead in order to facilitate transferring the child to the chair. The camera height change relative to the floor increased the field of vision (FOV) by 80% and a camera recalibration was required. The child who volunteered in the case study reported here had a severe limitation in her hand range of motion, and thus we moved the joystick from the side to the front of her body to facilitate the joystick handle grasping. Furthermore, me reduced the handle height by 50%, and the side to side range of motion of the joystick by 40%. The change of FOV and the reduced range of motion of the joystick required a change in the controller gains, meaning that the child did not experience the same control law as the children in the "Guidance" group, although it was quite similar. The increase in FOV facilitated more freedom around the line, and thus the child with impairment was able to experience larger errors than the non-disabled children.

### Experimental Protocol

**Figure 3:**
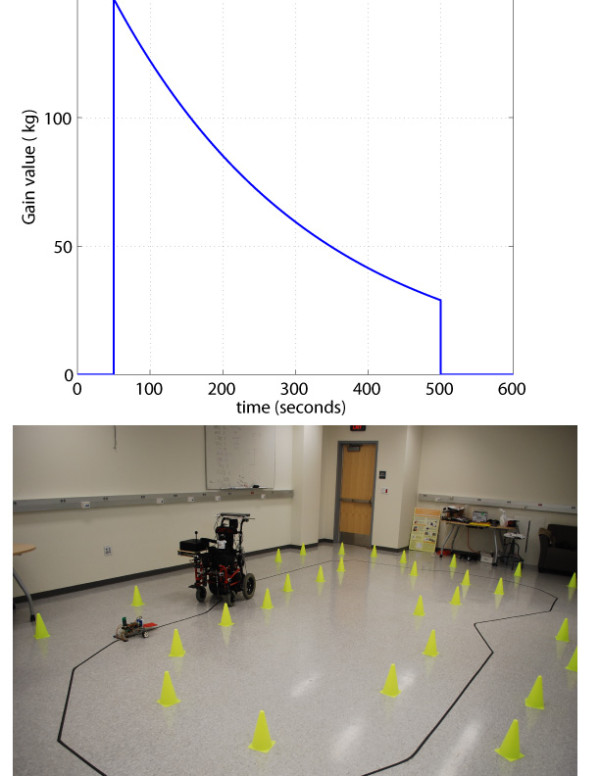
**Control gain and driving course**. Top: Control gain *K_j _*used for each subject. Subjects drove through the circuit during 50 seconds without robotic guidance from the robotic joystick followed by 450 seconds of robotically guided training, and 100 seconds without guidance. Down: Picture of the 19 m long driving course.

To determine if the robotic wheelchair trainer could help children learn to steer the wheelchair while limiting errors, we performed an experiment with 22 non-disabled children (aged 3-9, mean 6.6 ± 1.5 SD), and a child with a severe motor impairment due to cerebral palsy. All experiments were approved by the Institutional Review Board of the University of California at Irvine, and subjects provided informed consent. Non-disabled children were randomly assigned into two, age-matched groups of 11 members each. Children in the "No Guidance" group (average age 6.96 ± 1.33 SD) were instructed to drive without any guidance from the robotic joystick during 10 minutes, trying to keep a laser pointer (pointing to the ground just below the child's feet) on the black line that defined the 19 m long driving circuit (Figure 3, down). Children in the "Guidance" group (average age 6.43 ± 1.47 SD) drove during the first 50 seconds without robotic guidance, followed by 9 trials (450 seconds) with a form of guidance that was systematically decreased by reducing the joystick's mechanical impedance (Figure 3, Top), and two last trials of 50 seconds without guidance.

The child with a severe motor impairment who performed the experiment is a bright but severely physically impaired 8-year-old girl as a result of Cerebral Palsy at birth. She had low tone in her trunk and could not use her upper extremities well. She had not self-initiated mobility when very young, and she did not pass the cut off points on the Powered Mobility Readiness test [25] until she was 4 1/2. Initially she used switches to learn to drive her power wheelchair for the first few months to learn control of direction' as using a proportional joystick was too demanding and overwhelming with her processing impairments. At the time of the study she used a center mount proportional joystick to drive her power wheelchair at home. The child with the motor impairment replicated the single-session training protocol that the non-disabled children in the "Guidance" group participated in.

### Data and statistical analysis

One participant in the Guidance group did not finish the experiment because she felt afraid, so data was analyzed for 10 subjects only. At each time sample, the tracking error, speed of the chair, and the value of the guidance control gains *K_j _*and *B_j _*, were measured.

To determine whether the guidance reduced the tracking error and increased speed when it was first introduced, we performed a paired t-test in the Guidance group comparing the mean errors and speed in the first experiment's trial with the error created during the second trial (when guidance was first applied). We performed an independent samples t-test to compare the error created during trials between groups. To test the training effectiveness of the guidance strategy, we compared the tracking error and speed between the first trial and the last trial, which were both without guidance, through a paired t-test. To determine whether guidance improved learning compared to no guidance, we used an independent samples t-test to compare the final mean distance error between the two groups. We tested with an independent sample t-test if either of the two strategies was more effective at reducing errors from trial 1 to the last trial without guidance. We also tested with an independent sample t-test if the child with the motor impairment reduced errors from trial 1 to the last trial without guidance by an amount similar than the children without motor impairment, in any of the two guidance strategies. The significance level was set to 0.05 for all tests.

## Results

### Guidance significantly reduced tracking error and increased speed of non-disabled children when applied during training

Twenty-two non-disabled children (aged 3-9) attempted to drive the smart powered wheelchair trainer around a 19 m circuit defined by a black line, in order to catch a small mobile robot moving ahead of them along the line, in a game of "robot tag". The chair slowed if they moved too far away from the black line. Half of the children trained without any haptic guidance, while half experienced faded haptic guidance throughout the training laps. At the end of the training session, we measured improvements in unassisted line tracking error, compared to at the beginning of the training session.

**Figure 4:**
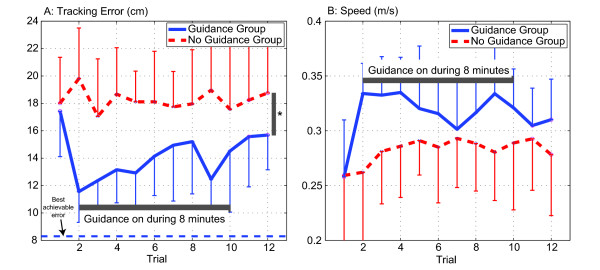
**Average tracking errors and mean speed during training of 22 non-disabled children aged 3-9**. Children in the Guidance group did not receive assistance on trials 1, 11 and 12, and received faded guidance during trials 2-10. Children in the No Guidance group did not receive assistance during training. A: Tracking error during each 50 s trial. Note that the tracking error was significantly reduced when guidance was applied at trial 2. When guidance was removed during the last 2 trials, children who trained with guidance followed the line better than children who never received guidance. B: Mean speed during each trial. Note the increase of speed in the guidance group when guidance was applied at trial 2. Error bars in all plots show ± 1 SD. *p < 0.05, t-test.

The robotic assistance provided by the smart wheelchair's robotic joystick was effective in reducing steering errors while it was applied, as evidenced by the fact that faded guidance reduced the tracking error on the first trial when guidance was applied, compared to the initial trial without guidance (Figure 4A, t-test, *p *< 0.001). It also resulted in better steering performance across the trials it was applied when compared to the no guidance group (individual trials 2-6, *p *< 0.01, and individual trials 7-10 not significant, *p *< 0.14). Similarly, the guidance increased the driving speed on the first trial when guidance was applied, compared to the initial trial without guidance (Figure 4B, t-test, *p *< 0.001), and resulted in faster driving across the initial trials it was applied when compared to the no guidance group (trials 2-4, *p *< 0.01). Because the guidance was faded gradually, when the robotic guidance was removed in trial 11, there was not a significant increment in error or decrement of speed when compared to the last trial with guidance.

### Training with guidance improved unassisted steering performance of non-disabled children

**Figure 5:**
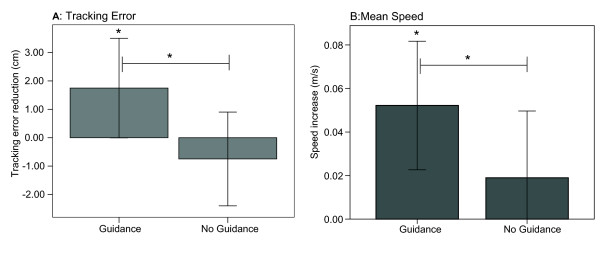
**Tracking error and speed increase from initial trial to last trial**. A: Non-disabled subjects in the Guidance group significantly reduced more the tracking error than subjects who trained without guidance. B: Non-disabled subjects in the Guidance group significantly increased the speed after training, and there was a non-significant tendency of a greater speed increase in the guidance group (*p *= 0.1413). Error bars in all plots show +/- 1 SD. *p < 0.05.

Non-disabled participants in the guidance group improved their unassisted steering performance following training with faded guidance. The guidance group showed better performance characterized by a significant reduction of the tracking error from trial 1 to last trial unassisted (trial 12) (Figure 5A, t-test, *p *= 0.05), and a significant increase of the driving speed (Figure 5B, t-test, *p *= 0.003). In the no guidance group, both the tracking error and driving speed remained without significant changes from trial 1 to last trial 12 (Figure 5A, B).

### Training non-disabled children with haptic guidance produced better performance at the end of the training session than non-guided training

Non-disabled participants who trained with physical guidance improved their steering performance more than subjects who trained without guidance. The faded guidance group showed a larger performance improvement characterized by a greater reduction of the tracking error from trial 1 to the last unassisted trial (trial 12) (Figure 5A, t-test, *p *= 0.031) compared to the non-guidance group, and a significant tendency of driving faster after training (Figure 5B, 1 tailed t-test, *p *= 0.05). The final tracking error (on trial 12) for the guidance group, was significantly less than the final tracking error for the group that learned without guidance (Figure 4A, t-test, *p *= 0.05). The guidance-trained group showed a faster speed after training, but the difference was not significant (Figure 4B, t-test, *p *= 0.1413).

### Effect of age on initial performance

**Figure 6:**
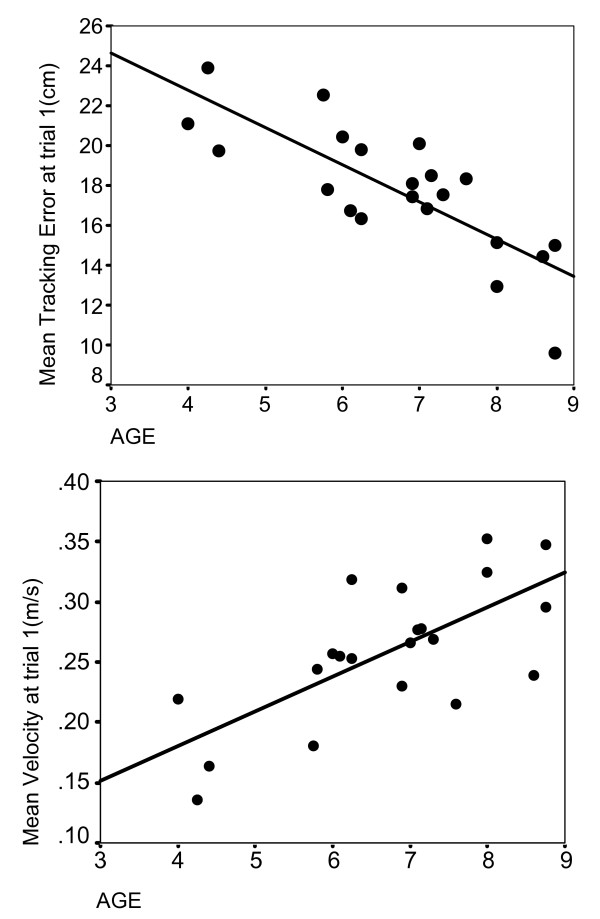
**Initial performance improves with age**. Top: There is a linear correlation between age and tracking error during trial 1. Down: Linear relationship between age and speed at trial 1.

We found a significant linear relationship between initial steering skill level and age. Very young children systematically performed worse than older children when steering the power wheelchair through the circuit, creating large errors and systematically losing the black line. Very young children especially had problems commanding the direction and speed of the wheelchair simultaneously, resulting in large tracking errors (Figure 6 top, Pearson's coefficient, *r *= 0.795, *p *< 0.001), and slower speed (Figure 6 bottom, Pearson's coefficient, *r *= 0.702, *p *< 0.001).

### A child with a severe motor impairment due to CP can benefit in the short-term from haptic guidance during driver's training

One 8-year-old child with a severe motor impairment due to cerebral palsy (CP) replicated the single-session training protocol performed by the non-disabled children in the "Guidance" group with small ergonomic changes of the system (see Methods). At the end of the training session, we measured the improvement in the non-assisted line tracking error, and compared it to the relative improvements of the non-disabled children in the "Guidance" and "No Guidance" groups.

**Figure 7:**
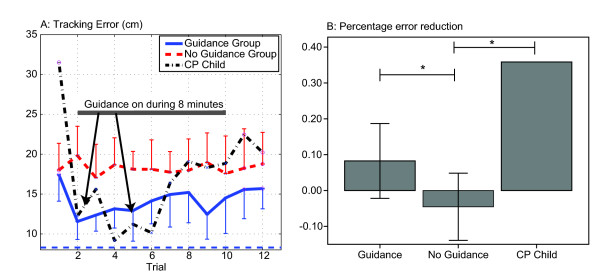
**Tracking errors during training of all subjects, 22 non-disabled children aged 3-9, and one 8-year-old child with a severe motor impairment due to cerebral palsy**. Children in the Guidance group and the child with CP did not receive assistance on trials 1, 11 and 12, and received faded guidance during trials 2-10. Children in the No Guidance group did not receive assistance during training. A: Tracking error during each 50 s trial. Note that the tracking error was significantly reduced when guidance was applied at trial 2 in both, non-disable children and child with CP. When guidance was removed during the last 2 trials, children who trained with guidance followed the line better than at the beginning of the training session. B: Percentage of tracking error reduction from trial 1 to last trial. The child with CP significantly reduced more the tracking error than children without a motor impairment who trained without guidance, and showed a tendency of larger reduction than children without a motor impairment trained with guidance (*p *= 0.104). Error bars in all plots show +/- 1 SD. *p < 0.05.

The tracking errors created by the child with CP during the training protocol follow a similar patter as those created by the non-disabled children in the "Guidance" group (Figure 7A). The error was reduced when the assistance was introduced in the second trial, and it increased systematically as the guidance was faded. When the guidance was removed, the tracking error remained smaller than the tracking error in first trial.

As described in the Methods section, we moved the webcam to an overhead location to facilitate the child with special needs sitting transfer. This change on the camera height increased the FOV, and thus allowed the child with cerebral palsy to experience larger errors around the line. Hence, it was not possible to compare the initial and final tracking errors between the child with CP and the non-disabled children. However, we found that the child with CP improved her steering ability after training with guidance from the joystick by a percentage greater than the children without motor impairment both in the "Guidance" group (Figure 7B, 1 sided t-test *p *= 0.05) and in the "No Guidance" group (Figure 7B, t-test, *p *= 0.02). There were no significant differences in the driving speed change from trial 1 to 12 between the child with CP and non-disable children in any guidance groups.

## Discussion

We developed a smart wheelchair on which young children can safely learn and develop driving skills at their own pace with minimum assistance from a therapist. We implemented a vision system able to detect a line on the floor, track it and calculate the position of the wheelchair with respect to the line. We also developed an algorithm that can demonstrate (through movements from a force feedback joystick) exemplary control to follow the course, while systematically modulating the strength and sensitivity of the haptic guidance. We designed an engaging training game using this technology, in which the driver tries to catch a small mobile robot moving ahead of him or her on the course.

In a pilot study with non-disabled children, we found that learning to drive a power wheelchair with faded guidance did not hamper learning, but indeed promoted leaning of the steering task, within a single training session. Furthermore, training with guidance was more effective than training without guidance. Final tracking errors in the guidance group were significantly lower than in the no guidance group. The guidance group showed a greater increase of speed than the no guidance group.

We also reported the results of a case study with one 8-year-old child with a severe motor impairment due to cerebral palsy trained with faded guidance. This child also improved steering ability after training with guidance from the joystick by an amount even greater than the children without motor impairment. We first discuss the implications of these results for wheelchair technology, motor learning research, and robot rehabilitation and then describe important directions for future research.

### Implications for wheelchair technology

A powered wheelchair offers a means of independent mobility to individuals with disabilities [26]. However, some individuals with severe disabilities lack the necessary motor control, or cognitive skills to easily learn to drive a wheelchair, and therefore have no other practical option for independent mobility [27]. Examples of such populations include children with cerebral palsy (CP), our first target population, but also people with high-level spinal cord injury (SCI), multiple sclerosis (MS), brain injury (BI), and stroke. To accommodate these individuals' mobility needs, there have been multiple attempts to develop "Smart Wheelchairs" (e.g. [1,8,26,28]). These technologies usually aim at providing fully or semi-autonomous navigation. However, provision of such a semi autonomous wheelchair could unintentionally prevent the development of new driving skills. Development of such skills could in turn simplify the technological requirements of the prescribed smart wheelchair, for example, by allowing chairs with obstacle avoidance but not advanced navigation computation and control, to be useful for more people.

The approach described in this paper is designed to lower the cost and improve accessibility to training for individuals with severe sensory motor impairments who require intensive/long duration practice to become competent in powered mobility. The technology we developed in this study could serve as an affordable way to allow individuals by themselves to attain some of the skills necessary to safely drive a standard powered wheelchair. We hypothesize that many people who are currently unable to drive a wheelchair can learn to drive in a structured environment given proper intensive training.

The joystick used in this study cost $4K. While this may be an acceptable cost for a training device that gets many hours of use by multiple users, it would be even more desirable to use a lower cost joystick. We have done preliminary evaluations on less expensive joysticks including the Microsoft Sidewinder Force Feedback and the The Novint Falcon. The Microsoft joystick proved to be too weak for the application, and the Falcon joystick's sensitivity was low and the communication speed two slow for fine control of the desired position of the handle. More research on how to adapt very low cost joysticks is needed.

We highlight that the focus of our work so far is learning to drive in a structured environment, which is an important first step for three reasons. First, learning to drive in a structured environment allows experience of dynamic self-initiated movement, a critical aspect of advancement of cognitive, perceptual, and motor abilities [1-4]. This technology has the potential to make the experience of dynamic self-initiated movement more widely accessible. Second, learning to drive in a structured environment in the clinic could enhance the use of smart wheelchair technology outside the clinic [1,8,26,28]. With simple modifications added to the home or school (e.g. a line on the floor between play areas), smart wheelchair technology would allow new driving skills to be used outside the clinic. Third, success at learning to drive in a structured environment is a necessary precursor for learning to drive independently in an unstructured environment.

### Implications for motor learning research

These results extend our previous findings [6,22] about the benefits of physical guidance for enhancing learning of a steering task. Previous work was performed using a virtual environment with adult subjects steering a robotic steering wheel. This work shows that haptic guidance provided by a joystick helps children develop a real-world steering skill.

As explained in [22] a possible interpretation of these results is that subjects performing complex tasks such as skiing [29], learning a complex spatiotemporal trajectory [10] and driving a vehicle learn to anticipate the timing of their movements better with cues provided by haptic guidance, such as the moment to begin a turn when encountering a sharp curve or the moment to rectify after a curve [6]. The concept that guidance can improve the learning of anticipatory timing is also consistent with the results of a recent experiment we performed [30], which showed a benefit of haptic guidance from a robot on less skilled participants in learning to play a time-critical task (pinball game). In the same line, recent work [10,31,32] found a benefit of haptic guidance from a robot in learning to reproduce the temporal, but not spatial, characteristics of a complex spatiotemporal curve. Thus, there is emerging evidence that haptic guidance may be specifically useful for learning anticipatory timing of forces in dynamic tasks. These results also have implications for the long-standing Guidance Hypothesis from motor learning research, which states that providing too much guidance will inhibit motor learning because it obviates the motor system from learning the necessary motor control strategies to perform the desired task. Since guidance was provided on all training laps during the steering training, the question arises why this continuously-provided guidance was not "too much", and thus did not inhibit learning. The possible negative effects of guidance may have been reduced because we used a compliant, faded form of guidance (cf. [13]). The amount of guidance decreased as training progressed, offering the driver the ability to overpower the joystick, which perhaps encouraged the user to pay attention and to develop appropriate motor control strategies. Alternately, the driving task itself may be peculiarly amenable to guidance-based training, and thereby forming an exception to the Guidance hypothesis, because it requires the learning of timing of forces.

Fundamentally, these results show that a simplistic interpretation of the Guidance Hypothesis - that guidance categorically impairs learning - misses an important aspect of human motor behavior: training with appropriately designed physical assistance can enhance the ability of the brain to learn some motor skills. The mechanisms of this benefit are still unclear. One possibility is that guidance may demonstrate better movement strategies (such as the need to initiate turns earlier). Alternately, guidance may make difficult tasks more optimally challenging, and thus improve motor learning, as suggested by the Challenge Point Theory [9]. Haptic guidance may make the input-output relationship between joystick motion and wheelchair motion more intelligible to the youngest children, preventing the chair from wandering too far from the path, in which case complex joystick motions are needed to return to the path.

These hypotheses may help to explain the surprising finding that the nondisabled children did not become better at driving the wheelchair after training without guidance. Of course, given a longer training time, we believe it is likely that they would have improved their performance. However, even with the short training duration, the group that received guidance improved their performance. This indeed suggests that the non-guided group was perhaps stuck in a "local minimum", in which they rapidly (within the first lap) became adequate at driving, but could not figure out how to improve further. As hypothesized above, the group that trained with guidance may have learned from the haptic demonstration of more skilled driving, or may have experienced a task that was more appropriately challenging because of the haptic guidance, allowing them to learn more quickly.

Another interesting aspect of the results described here is that the tracking error increased (and speed was reduced) during the last few trials with guidance (Figure 4, trials 6-8), when the guidance has already been faded by more than 70% of its initial value (Figure 3). The faded guidance algorithm defined in Equation 3 was independent of the participant's performance level. Because not everybody learns at the same rate, this increase in tracking error might be due to an early excessive reduction of the assistance in some unskilled subjects. An adaptive fading algorithm (such as the one described in [6]), that systematically reduces the guidance applied to the driver based on real-time measurement of tracking performance, may have better limited the amount of tracking errors during all trials where guidance was applied. Such a "Guidance-as-needed" algorithm would slowly decrease the assistance on the drivers hands when tracking error is small, but would increase the assistance in response to larger tracking errors.

### Implications for robot rehabilitation research

We reported the results of a case study with one 8-year-old child with a severe motor impairment due to cerebral palsy trained with faded guidance. This child also improved steering ability after training with guidance from the joystick by an amount even greater than the children without motor impairment. This case study indicates that a child with a impaired motor system can also benefit from haptic guidance during driver's training, like a child with a non-impaired motor system, at least in a single training session. This finding suggests that normative motor learning mechanisms will continue to work in impaired motor systems.

A secondary result from this study might reinforce the idea that guidance enhance motor learning in impaired motor system. We did not find a significant difference in the driving speed change from trial 1 to 12 between the child with CP and the non-disabled children in any guidance groups. The child with CP (and very young children) especially had problems commanding the direction and speed of the wheelchair simultaneously, resulting in large tracking errors and a slow motion. Apparently, the child with CP did not benefit from the haptic guidance to increase the steering speed, probably because guidance was applied only in the joystick x-axis (to control steering), while the joystick y-axis (to control speed) was entirely controlled by the driver. Thus, the child with CP learned to perform better only the task where guidance was directly applied (steering), while no difference was observed on the side task where guidance was missing (speed). We hypothesize, that applying guidance also in the joystick y-axis may enhance learning of commanding the wheelchair speed.

The study reported here was conducted mainly with non-disabled participants in a single training session. We chose to first study non-disabled children in a single session partly for convenience, but also because it is important to establish the normative learning mechanisms of the non-injured motor system, thereby providing a framework for comparison for future studies with children with a disability. Future work will focus on testing with a larger group of children with a disability to determine if children with a motor impairment consistently learn in a similar way. We speculate that normative motor learning mechanisms will continue to work in children with motor impairments, but in some case children with motor impairments may require longer periods of practice, with guidance reduced based on ongoing performance, to achieve optimal motor learning benefits.

### Other Future Directions

Another result from the present study that is encouraging looking forward to this future work relates to the fact that there was a significant linear relationship between initial steering skill level and age. Very young children systematically performed worse than older children when steering the power wheelchair thought the circuit, creating large errors and losing the black line many times. In previous work with the wheelchair simulator we found that haptic guidance was especially beneficial for less skilled subjects [22]. Similarly, in [30], initially less skilled participants exhibited better learning of a pinball task when trained with physical guidance. Since the wheelchair is ultimately intended for severely disabled, very young children, and the initial performance of these children is likely poor, the finding that guidance is more beneficial for less skilled participants is encouraging.

The ability to drive a wheelchair independently requires more than the ability to track a line. One possibility is to incorporate physical doorways and ramps on parts of the course to work on developing skills in negotiating common physical environments. Because the trainer chair will be equipped with line-following and obstacle proximity sensors, training can be made safe. Another option is to develop a "free play mode" in which the user can practice steering without haptic assistance in a "safe" encircled area defined by a colored line on the floor, but turning around the wheelchair when the vision system detects that the child is trying to leave the "safe" area. Ultimately, we envision creating a training experience that compares favorably with the fun children experience with the best amusement park rides, but that facilitates the development of driving skill.

## Competing interests

The authors declare that they have no competing interests.

## Authors' contributions

LMC designed and developed the smart wheelchair system, run the study, performed the statistical analysis and draft the manuscript. JF participated in the design of the experimental setup with the child with disability, helped in the recruitment of subjects, and helped to improve the system to accommodate children with special needs. DJR and JF contributed concepts and edited and revised the manuscript. All authors read and approved the manuscript.

## Consent

Written informed consent was obtained from the patient for publication of this case report and accompanying images. A copy of the written consent is available for review by the Editor-in-Chief of this journal.
